# Chemokine-Ligands/Receptors: Multiplayers in Traumatic Spinal Cord Injury

**DOI:** 10.1155/2015/486758

**Published:** 2015-04-21

**Authors:** Friederike Knerlich-Lukoschus, Janka Held-Feindt

**Affiliations:** Department of Neurosurgery, University Medical Center Schleswig-Holstein, Arnold-Heller-Strasse 3, Haus 41, 24105 Kiel, Germany

## Abstract

Spinal cord injury (SCI) results in complex posttraumatic sequelae affecting the whole neuraxis. Due to its involvement in varied neuromodulatory processes, the chemokine-ligand/receptor-network is a key element of secondary lesion cascades induced by SCI. This review will provide a synopsis of chemokine-ligand/receptor-expression along the whole neuraxis after traumatic spinal cord (sc) insults on basis of recent in vivo and in vitro findings in a SCI paradigm of thoracic force-defined impact lesions (Infinite Horizon Impactor) in adult rats. Analyses of chemokine-ligand/receptor-expression at defined time points after sc lesion of different severity grades or sham operation revealed that these inflammatory mediators are induced in distinct anatomical sc regions and in thalamic nuclei, periaqueductal grey, and hippocampal structures in the brain. Cellular and anatomical expression profiles together with colocalization/expression of neural stem/progenitor cell markers in adult sc stem cells niches or with pain-related receptors and mediators in dorsal horns, dorsal columns, and pain-processing brain areas support the notion that chemokines are involved in distinct cascades underlying clinical posttraumatic impairments and syndromes. These aspects and their implication in concepts of tailored SCI treatment are reviewed in the context of the recent literature on chemokine-ligand/receptor involvement in complex secondary lesion cascades.

## 1. Introduction

Spinal cord injury (SCI) continues to pose serious clinical and socioeconomic problems given that affected individuals often face a multifaceted spectrum of complex long-term sequelae including loss of motor function, vegetative dysfunction, development of central pain syndromes, and even cognitive impairment [1, 2]. Despite considerable progress in primary and rehabilitative care, curative therapies remain elusive to date resulting in unsatisfactory management and poor outcome of spinal cord injured patients with considerable negative impact on their psychosocial life [3].

One prerequisite for developing tailored mechanism-driven therapies is a detailed understanding of pathological cascades and cellular changes that are induced by the initial trauma. These cascades, which are subsumed under the umbrella term “secondary lesion,” have been subject of intense research over the last decades. Thereby, posttraumatic inflammation was identified as crucial component of these mechanisms, with inflammatory mediators and associated reactions becoming evident in the acute, subacute, and late chronic posttraumatic course after SCI, which promote or inhibit neuroregenerative processes [4–7]. With regard to clinical settings, these mechanisms therefore bear practicable therapeutic potential because tailored SCI medical treatment will be feasible only after primary stabilization of the affected individuals.

In terms of factors mediating inflammatory reactions after SCI, the chemokine-ligand/receptor-system is of particular interest. Due to their widespread induction after SCI and their versatility, one can assume that these chemotactic cytokines and their receptors bear promising potential for future mechanism-driven trials.

## 2. Mediators of Secondary Inflammatory Reactions: Chemokines and Their Receptors

**Figure 1:**
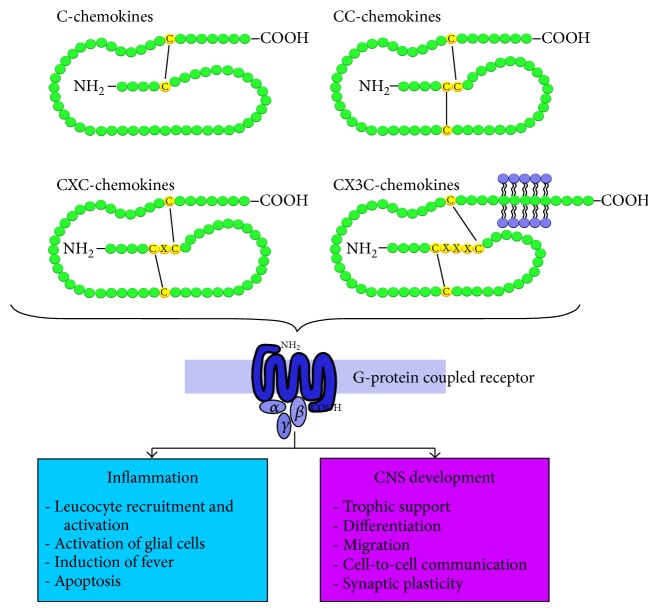
Chemokine classification. Based on the presence of a cysteine motif in the N-terminal region of the protein, chemokines divide into four subgroups (alpha-, beta-, gamma-, and delta-chemokines). Through activation of G-protein coupled chemokine-receptors, these chemotactic proinflammatory mediators are involved in varied pathological and physiological processes.

Chemokines and their receptors are important versatile components of varied secondary inflammatory mechanisms after different neurological damages. These chemotactic proteins of low molecular weight (8 kDa to 12 kDa) are involved in a wide spectrum of physiological and pathological processes, also involving the CNS [8–13]. Based on their biochemical structure, that is, presence of a cysteine motif in the N-terminal region of the protein, they divide into four subgroups (alpha-, beta-, gamma-, and delta-chemokines) and act on G-protein coupled chemokine-receptors [14] (Figure 1).

Chemokines are key players in the hematopoietic and lymphatic system, including stem cell maturation, angiogenesis, and recruiting leucocytes to inflammatory foci. Beyond their “classical” inflammatory functions, chemokine expression was also described in the central nervous system (CNS). Here, chemokines were found to be coexpressed with cholinergic and dopaminergic neurotransmitters in neuronal cells of specific anatomical brain regions and identified as important cell-to-cell communicators [15]. Based on the concept that distinct anatomical distribution in exclusive brain regions is connected to specific functions, a chemokine-effector/receptor-system was postulated as “third transmitter,” beside the classical neurotransmitter and neuropeptide system in the brain [16].

Furthermore, chemokines and their receptors are also involved in neurodevelopmental processes [17–19]. CXCL12/CXCR4 especially was identified as an essential factor regulating secretion of neurotrophic factors, differentiation, and correct migration of neural stem/progenitor cells [20–22].

In principle, this multifunctionality makes the chemokine-ligand/receptor-system an attractive candidate for tailored SCI-therapeutic approaches which enable addressing varied aspects of the complex secondary lesion cascades and resulting clinical problems. Regarding their potential clinical relevance, different cytokines and chemokines (like IL-1, IL-8, MCP1, IL-16, and TNF) have been found on elevated levels in sera or cerebral spinal fluid of spinal cord injured patients [23–27]. As concrete example, Hassanshahi et al. recently reported increased CXCL1 and CXCL12 expression levels immediately after SCI (within 3–6 h), elevated CXCL12, CXCL1, CXCL9, and CXCL10 levels at 7 days, and prolonged CXCL12 expression up to 28 days after SCI [28].

This mirrors findings reported in animal SCI paradigms, which over all demonstrated that chemokines and cytokines are induced after different neurological trauma modalities [29] and are involved in secondary lesion cascades at different stages and CNS regions after SCI ([30, 31], and own investigations).

**Figure 2:**
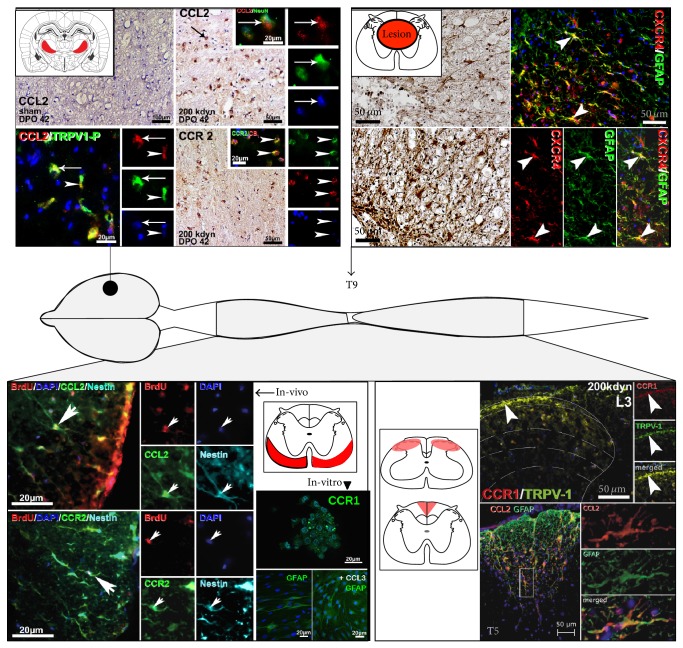
Chemokine-ligand/receptor-expression after impact SCI in adult rats. After impact lesions, different chemokines are induced in distinct anatomical regions along the whole neuraxis. This overview exemplarily illustrates chemokine-ligand/receptor-immunostaining along the neuraxis (diaminobenzidine and double/triple-immunofluorescence labeling). Schemes indicate anatomical location. On brain level, CCL2/CCR2 expression was found induced in thalamic nuclei in the late time-course, that is, 42 hours after severe SCI, which was colocalized with TRPV1 and CB1. Sham animals did not exhibit chemokine expression in this location. On lesion level T9 chemokine-ligands/receptors were induced in inflammatory cells in the early and astroglial cells in the later time-course. Furthermore, chemokines were induced in the ventrolateral white matter along the whole spinal cord. Here, chemokines colocalized with proliferating BrdU-labeled cells, which coexpressed astroglia and NPC markers (shown for nestin). In vitro, CCR1 was found consistently expressed on endogenous NPCs (demonstrated for a sham-derived neurosphere). Applying CCL3 to NPCs during differentiation cycles led to significant higher GFAP-mRNA and immunohistochemical level as compared to untreated neurosphere cultures (demonstrated for sham-derived cultures). Another anatomical location of chemokine induction was pain-related spinal cord areas like dorsal horns and dorsal columns. Here, chemokine expression colocalized among others with TRPV1 and glial markers. Details are discussed in the respective sections.

In a SCI paradigm of thoracic force-defined spinal cord impact lesions which were applied with the Infinite Horizon Impactor (Precision System and Instrumentation, Lexington, KY) [32] in adult male Long-Evans rats, chemokine-ligands and receptors were induced in distinct anatomical areas along the whole neuraxis, including spinal cord and brain regions in a lesion- and time-dependent manner [33–35] (Figure 2). Regional chemokine expression thereby correlated with changes in locomotor function and pain-related behavior. These findings will be summarized in light of the recent literature illustrating chemokine-ligand/receptor involvement in the complex intermingling cascades of secondary lesion after SCI.

## 3. Chemokine-Ligand/Receptor-Expression after SCI in the Central Nervous System

### 3.1. Chemokine Expression on Spinal Cord “Lesion Level”

SCI induces a strong early inflammatory response on the affected spinal cord level. For instance, cytokines and chemokines like TNF-1alpha, IL-1beta, CCL2, and CCL3 are found to be induced on mRNA and immunohistochemical levels within the first hours after spinal cord damaging incidences in close proximity or directly in the lesion core [36–38].

In the acute and subacute phase, cytokines and chemokines thereby colocalize with markers for monocytic and astroglial cells around the lesion core. In the chronic phase after impact SCI, chemokine-effector/receptor-expression remained significantly elevated on mRNA, protein, and immunohistochemical level, with C-C- and C-X-C-chemokine-effectors/receptors coexpressed or colocalized in GFAP-labeled activated astrocytes, which over time formed a gliotic wall around the developing posttraumatic syrinx [33] (Figure 2; lesion level).

On the one hand, astrocytes are known to form these protecting barriers around damaged CNS areas thus shielding off surrounding tissues from damaging factors [39]. On the other hand, astrocytes themselves seem to maintain inflammation through chemokine production and recruitment of inflammatory cells [40], which together with further upregulated inhibitory molecules in the scar tissue presuppose an antiregenerative milieu in the lesion rim [41].

Various anti-inflammatory therapy options to balance destructive and reparative functions of proinflammatory cascades have been developed but so far did not prove to be effective in the clinical routine (for review [42]).

### 3.2. Chemokine Enrollment in Remote Spinal Cord Regions: Chemokine-Ligand/Receptor-Expression in the White Matter Spinal Cord Neural Stem/Progenitor Cell Niche

Local induction of a lesion or trauma in the spinal cord leads to changes along the whole spinal axis, and chemokines are one of the underlying mediators that seem to perpetuate these mechanisms. After experimental local thoracic impact lesions, these molecules and their receptors are induced on mRNA, protein, and immunohistochemical level not only in the lesion itself, but also, additionally far beyond, in the lumbar, higher thoracic, and cervical spinal cord [35, 43].

Thereby, CCL2/CCR2, CCL3/CCR1, and CXCL12/CXCR4 were also found in significantly elevated level in the ventrolateral white matter, a recognized neural stem/progenitor cell niche of the adult spinal cord [44–46]. Here, chemokine-effector/receptor-immunoreactivities were coexpressed with astroglial (GFAP), radial glial (BLBP, 3CB2), and neural progenitor cell (nestin, musashi) and in part oligodendroglial (CNPase) markers (Figure 2; ventrolateral white matter).

This anatomical region exhibited significantly elevated subpial cell proliferation, confirmed with BrdU-labeling, which peaked in the first days after lesion and remained significantly elevated late into the chronic time-course after SCI [43]. BrdU-labeled cells were colocalized with neural progenitor cell (NPC) markers like nestin, musashi-1, NG2, and radial glia markers like BLBP and 3CB2 which in turn were partially colocalized or coexpressed with CCL2/CCR2, CCL3/CCR1, and CXCL12/CXCR4 (Figure 2).

These findings suggested that after SCI chemokines and their receptors might become crucial determinants of endogenous NPCs' microenvironment, a notion that was supported by further in vitro investigations [47]. NPCs which were isolated from thoracic spinal cord segments of adult rats after receiving severe thoracic impact SCI (200 kdyn) or sham-laminectomy and which were cultured according to established protocols for spinal cord-derived NPCs [45, 46] exhibited consistent CCR1-mRNA and corresponding immunohistochemical expression (Figure 2). After differentiation, this CCR1-mRNA expression decreased to significant lower levels compared to expression levels in neurosphere cultures. Applying “external” CCL3—the main ligand of CCR1—to neurosphere cultures during their differentiation cycles led to significant elevated GFAP-mRNA amounts in differentiated sham-derived cell cultures.

Unlike in sham-derived cell cultures, pretreatment of CCL3 had no significant effect on glial differentiation in spinal cord lesion cultures [47], which could be a preconditioning effect on lesioned-derived NPCs that already faced an altered microenvironment after SCI application before being isolated for cell culture. As outlined above, secondary inflammatory processes with expression of respective mediators are induced within the first hours after SCI and consequently alter the cellular microenvironment. In this way, NPCs were most likely already exposed to diverse chemokines and cytokines, which in turn may desensitize respective receptors [48].

Such preconditioning processes can be assumed to also underlie another in vitro observation. After running though differentiation cycles with growth factor restriction, NPCs differentiate into the three major cell lines with high proportion of GFAP-immunohistochemically labeled cells and beta-III tubulin- and CNPase-positive cells. Thereby, NPCs derived from lesion animals exhibited significant higher GFAP-mRNA-expression level compared to sham control cultures [47].

Both in vivo and in vitro findings support the notion that the chemokine-ligand/receptor-system plays a specific role in the microenvironment of endogenous NPCs, especially in astroglial/radial glial differentiation. Further hints for C-C-chemokine involvement in astroglial differentiation were provided by Lawrence et al. [49], who demonstrated CCL2 upregulation through NF-*κ*B and NF-iB during progenitor cell differentiation towards astrocytic phenotypes.

Thereby, radial cells are viewed as pluripotent neural precursors in the adult CNS and are important among others in guiding migrating neurons, regulating axon outgrowth and pathfinding during white matter patterning [50–54]. After lesional incidences, radial glial cells have been reported to support neuroregenerative processes among others by forming bridges across the lesion side and providing protecting barriers against neurodestructive elements [55, 56]. Regarding other chemokine-ligand/receptor-systems, the CXC-chemokine CXCL12, which was recently shown to be released by radial glial cells, coordinates neuroblast migration during vessel growth and patterning in human fetal brains [57].

Further investigations of intracellular signal transduction pathways are required for a deeper understanding of different chemokines' impact on endogenous NPCs and involvement in neuroregenerative processes after SCI. However, as outlined in the previous section, excessive gliosis also has its negative aspects; balancing potential beneficial and destructive/inhibitory effects of postinflammatory mechanisms poses therefore major challenges in developing anti-inflammatory therapies. Hereby, addressing time-specific changes might provide a clue in future functional trials, promoting or inhibiting different chemokine-ligand/receptor-systems.

### 3.3. SCI-Induced Chemokine-Ligand/Receptor-Expression in Dorsal Horns and Dorsal Columns: Impact on Development of Posttraumatic Neuropathic Pain

Involvement of chemokines as pronociceptive factors in pain development especially in response to peripheral nerve lesion is well recognized (for review [58–63]). For example, chemokine expression and possible functional roles in dorsal horns pain-related behavior have been reported in detail for fractalkine CXCL1 and its receptor CX3CR; Verge et al. demonstrated CXCL1 as a neuron-to-glia messenger during high levels of neural stimulation that was associated with nociceptive transmission [64]. Thereby, cathepsin S, a lysosomal enzyme involved among others in nociceptive processes, when released by microglia was shown to be responsible for cleavage of the CX3CL1 from neuron membranes [65, 66]. CX3CL1 and its receptor were also implicated in the sensorial changes associated with other inflammatory disease associated polyradiculopathies such as Guillain Barré syndrome [67, 68].

Own comprehensive anatomical analyses of time-dependent C-C- and CXC-chemokine expression patterns after different SCI impact lesion grades in conjunction with behavioral testing supported the notion that these chemokines are also crucial factors in neuropathic pain development after central SCI. CCL2/CCR2, CCL3/CCR1, and CXCL12/CXCR4 were additionally expressed in a lesion- and time-dependent manner in the superficial layers of spinal cord dorsal horns and dorsal columns [33, 35] (Figure 2; dorsal horns and dorsal columns). These expression patterns correlated significantly with the development of pain related behavior. Dorsal horn chemokine induction was found in all lesion treatment groups in the early post-SCI time course in which animals of all lesion grades exhibited acute mechanical hypersensitivity. In the late time-course after SCI, only animals that received severe lesions exhibited persistent below-level pain correlating (mechanical and thermal hypersensitivity) with prolonged dorsal horn chemokine expression. These findings suggested that chemokine-ligands/receptors-mediated mechanisms time dependently underlie different pain entities like acute, inflammatory pain in the early time course and prolonged central pain in the late survival time course.

Thereby, dorsal horn chemokine expression in our SCI paradigm was coexpressed with astroglial marker (CLL2/CCR2, GFAP) [33] and neuronal marker (CCL3, NeuN) [35], which is—notwithstanding controversies regarding cellular chemokine-receptor localization in different experimental paradigms [61]—in concordance with the current concept of cytokine mediated neuron-nonneuronal cell communication in neuropathic pain development [69, 70]. As such, astrocytes are now viewed as potential key players in chronic neuropathic pain development (for review [71]).

Colocalization or coexpression of chemokines with peptides, neurotransmitters, and receptors involved in nociception, such as transient receptor potential vanilloid receptor (TRPV-1), Calcitonin Gene Related Peptide (CGRP), and substance-P, in dorsal horn laminae I and II, and dorsal columns provide further evidence of chemokine involvement in sensitivity-modulating processes after SCI.

While trials addressing therapeutic implications (antibodies/antagonists directed against and pharmacological inhibition of chemokine-ligand/receptor-signaling; intrathecal delivery of chemokine antibodies or systemic treatment with chemokine-receptor antagonists) further supported the notion that chemokines are involved in neuropathic pain development and showed some efficiency in animal models, translation into clinical settings failed to proof efficacy so far and remains to be a challenging ongoing task (for review [61]).

### 3.4. Induction of the Chemokine-Ligand/Receptor-System in Remote CNS Regions: Chemokine Involvement in SCI-Induced Changes on Brain Level

Since individuals suffering from SCI often face problems like central pain development, fatigue, and cognitive impairment in the chronic phase after the trauma [1], changes on supraspinal level were included in investigations addressing secondary lesion mechanisms after SCI. In this sense, complex alterations on brain level like thalamic somatotopic reorganization [72] and thalamic microglial activation with CCL21 induction [73] have been shown to possibly underlie central pain development after SCI.

As outlined above, chemokine-ligands/receptors are also expressed in the brain and are important regulators of different physiological and pathological processes. After application of impact lesions in our SCI paradigm, C-C-chemokines CCL2 and CCL3 as well as their receptors were induced lesion grade and time dependent in distinct brain regions [34]. As such, constitutive hippocampal CCL2/CCR3 and CCL3/CCR1 immunohistochemical expression in the CA1/CA3-stratum pyramidale and granular and polymorph cellular layers of the dentate gyrus were induced and colocalized with significant elevated cannabinoid-receptor 1- (CB1-) immunoreactivity.

Further chemokines were induced in thalamic nuclei (reticular thalamic nucleus, ventral posterolateral thalamic nucleus, ventral posteromedial thalamic nucleus), which did not exhibit constitutive expression patterns (Figure 2). Here, CB1 was found on reduced level in the long-term after severe spinal cord lesions. Considering a further pain related anatomical region, CCL2/CCR2 and CCL3/CCR1 were found to be induced in in the early phase (first 7 days after SCI) after moderate (100 kdyn) and severe (200 kdysn) lesions while in the late time course (42 days after SCI) elevated chemokine levels were only found after severe SCI. Again, associated CB1 immunoreactivity followed an opposite expression pattern with significant reduction in both lesion groups at the earlier survival time points and prolonged reduced CB1 immunoreactivity densities only after severe lesion at 42 days after SCI. These findings mirrored behavioral changes as only 200 kdyn lesion animals exhibited prolonged lowering of their mechanical and thermal sensitivity thresholds—a correlate for neuropathic pain development—while 100 kdyn lesioned animals only exhibited acute hypersensitivity in the first days after SCI [33].

These findings suggested an involvement of the chemokine-ligand/receptor and cannabinoid system in central brain alterations after thoracic impact SCI. Thereby, the involved brain regions are associated with central pain development and cognitive, stress-related processes [74, 75], which probably underlie delayed neuropsychological sequelae observed in the delayed clinical course after SCI.

Interaction of the chemokine network with the cannabinoid system has been demonstrated in the PAG [76, 77] and is thought to take place through G protein-coupled heterologous receptor desensitization [78, 79].

In regard to pain processing, C-C-chemokine-ligands/receptors and CB1 were in part colocalized with phosphorylated TRPV1 in pain-relevant brain areas which supposes that heterologous desensitization and cross-sensitization may play a role in central processes underlying neuropathic pain development after SCI with concomitant strong inflammatory reactions [80, 81].

Considering these findings of chemokine induction in brain regions, local SCI induces changes far beyond the initially affected spinal cord level and also involves distinct brain regions and systemic organ systems [82], stressing out the complexity of secondary lesion cascades after SCI which in turn cannot be addressed with one single therapeutic approach.

## 4. Summary and Conclusion

**Figure 3:**
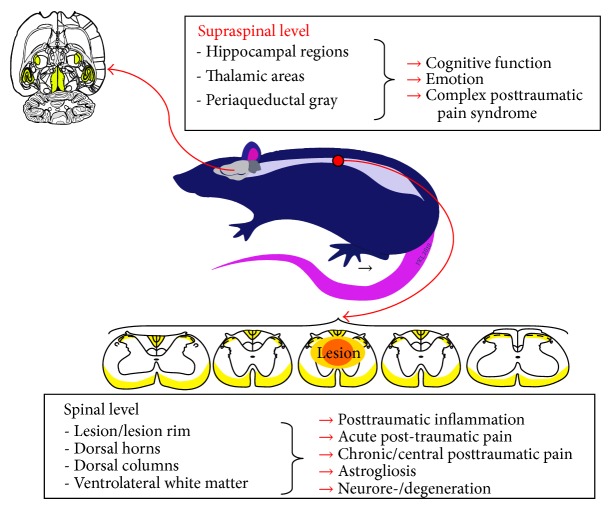
The chemokine-ligand/receptor-network is potentially involved in varied processes after SCI, making these mediators interesting targets for tailored therapeutic trials.

Chemokine-ligands/receptors are crucial mediators of inflammatory secondary lesion cascades after SCI. These multifunctional molecules are induced in time and lesion grade-dependent manner in distinct anatomical areas along the whole neuraxis (Figure 3). Hereby, cellular and anatomical expression profiles suggest that chemokine-ligands/receptors are involved in distinct cellular cascades depending on their induction time point and anatomical localization. These findings support the notion that from early on these molecules are involved as primary proinflammatory mediators in rather destructive secondary mechanisms in the lesion core and lesion rim but may in the later time-course support differentiation of distinct NPC in the ventrolateral white matter and maintain cellular processes on spinal and brain level underlying central pain development during the chronic phase after SCI. Thus, addressing the chemokine-ligand/receptor-network in clinical therapeutic trials is complex, and translation of preclinical evidence into effective SCI treatment options remains a difficult undertaking.

## References

[1] Jensen M. P., Kuehn C. M., Amtmann D., Cardenas D. D. (2007). Symptom burden in persons with spinal cord injury. *Archives of Physical Medicine and Rehabilitation*.

[2] Singh A., Tetreault L., Kalsi-Ryan S., Nouri A., Fehlings M. G. (2014). Global prevalence and incidence of traumatic spinal cord injury. *Clinical Epidemiology*.

[3] Rowland J. W., Hawryluk G. W. J., Kwon B., Fehlings M. G. (2008). Current status of acute spinal cord injury pathophysiology and emerging therapies: promise on the horizon. *Neurosurgical Focus*.

[4] Alexander J. K., Popovich P. G. (2009). Neuroinflammation in spinal cord injury: therapeutic targets for neuroprotection and regeneration. *Progress in Brain Research*.

[5] Donnelly D. J., Popovich P. G. (2008). Inflammation and its role in neuroprotection, axonal regeneration and functional recovery after spinal cord injury. *Experimental Neurology*.

[6] Jones T. B., McDaniel E. E., Popovich P. G. (2005). Inflammatory-mediated injury and repair in the traumatically injured spinal cord. *Current Pharmaceutical Design*.

[7] Trivedi A., Olivas A. D., Noble-Haeusslein L. J. (2006). Inflammation and spinal cord injury: infiltrating leukocytes as determinants of injury and repair processes. *Clinical Neuroscience Research*.

[8] Asensio V. C., Campbell I. L. (1999). Chemokines in the CNS: plurifunctional mediators in diverse states. *Trends in Neurosciences*.

[9] Tran P. B., Miller R. J. (2003). Chemokine receptors: signposts to brain development and disease. *Nature Reviews Neuroscience*.

[10] Tran P. B., Ren D., Veldhouse T. J., Miller R. J. (2004). Chemokine receptors are expressed widely by embryonic and adult neural progenitor cells. *Journal of Neuroscience Research*.

[11] Cartier L., Hartley O., Dubois-Dauphin M., Krause K.-H. (2005). Chemokine receptors in the central nervous system: role in brain inflammation and neurodegenerative diseases. *Brain Research Reviews*.

[12] Mennicken F., Maki R., de Souza E. B., Quirion R. (1999). Chemokines and chemokine receptors in the CNS: a possible role in neuroinflammation and patterning. *Trends in Pharmacological Sciences*.

[13] Williams J. L., Holman D. W., Klein R. S. (2014). Chemokines in the balance: maintenance of homeostasis and protection at CNS barriers. *Frontiers in Cellular Neuroscience*.

[14] Kuang Y., Wu Y., Jiang H., Wu D. (1996). Selective G protein coupling by C-C chemokine receptors. *Journal of Biological Chemistry*.

[15] Banisadr G., Gosselin R.-D., Mechighel P., Rostène W., Kitabgi P., Parsadaniantz S. M. (2005). Constitutive neuronal expression of CCR2 chemokine receptor and its colocalization with neurotransmitters in normal rat brain: functional effect of MCP-1/CCL2 on calcium mobilization in primary cultured neurons. *Journal of Comparative Neurology*.

[16] Adler M. W., Rogers T. J. (2005). Are chemokines the third major system in the brain?. *Journal of Leukocyte Biology*.

[17] Padovani-Claudio D. A., Liu L., Ransohoff R. M., Miller R. H. (2006). Alterations in the oligodendrocyte lineage, myelin, and white matter in adult mice lacking the chemokine receptor CXCR2. *Glia*.

[18] Tsai H.-H., Frost E., To V. (2002). The chemokine receptor CXCR2 controls positioning of oligodendrocyte precursors in developing spinal cord by arresting their migration. *Cell*.

[19] Tsai H. H., Frost E., To V. (2002). The chemokine receptor CXCR2 controls positioning of oligodendrocyte precursors in developing spinal cord by arresting their migration. *Cell*.

[20] Luo Y., Cai J., Xue H., Miura T., Rao M. S. (2005). Functional SDF1alpha/CXCR4 signaling in the developing spinal cord. *Journal of Neurochemistry*.

[21] Luo Y., Xue H., Pardo A. C., Mattson M. P., Rao M. S., Maragakis N. J. (2007). Impaired SDF1/CXCR4 signaling in glial progenitors derived from SOD1^G93A^ mice. *Journal of Neuroscience Research*.

[22] Dziembowska M., Tham T. N., Lau P., Vitry S., Lazarini F., Dubois-Dalcq M. (2005). A role for CXCR4 signaling in survival and migration of neural and oligodendrocyte precursors. *Glia*.

[23] Bank M., Stein A., Sison C. (2015). Elevated circulating levels of the pro-inflammatory cytokine macrophage migration inhibitory factor in individuals with acute spinal cord injury. *Archives of Physical Medicine and Rehabilitation*.

[24] Davies A. L., Hayes K. C., Dekaban G. A. (2007). Clinical correlates of elevated serum concentrations of cytokines and autoantibodies in patients with spinal cord injury. *Archives of Physical Medicine and Rehabilitation*.

[25] Kwon B. K., Stammers A. M. T., Belanger L. M. (2010). Cerebrospinal fluid inflammatory cytokines and biomarkers of injury severity in acute human spinal cord injury. *Journal of Neurotrauma*.

[26] Liu S.-Q., Ma Y.-G., Peng H., Fan L. (2005). Monocyte chemoattractant protein-1 level in serum of patients with acute spinal cord injury. *Chinese Journal of Traumatology*.

[27] Stein A., Panjwani A., Sison C. (2013). Pilot study: elevated circulating levels of the proinflammatory cytokine macrophage migration inhibitory factor in patients with chronic spinal cord injury. *Archives of Physical Medicine and Rehabilitation*.

[28] Hassanshahi G., Amin M., Shunmugavel A. (2013). Temporal expression profile of CXC chemokines in serum of patients with spinal cord injury. *Neurochemistry International*.

[29] Ransohoff R. M. (2002). Chemokines in neurological trauma models. *Annals of the New York Academy of Sciences*.

[30] Ghirnikar R. S., Lee Y. L., Eng L. F. (2001). Chemokine antagonist infusion promotes axonal sparing after spinal cord contusion injury in rat. *Journal of Neuroscience Research*.

[31] Taylor A. R., Welsh C. J., Young C. (2014). Cerebrospinal fluid inflammatory cytokines and chemokines in naturally occurring canine spinal cord injury. *Journal of Neurotrauma*.

[32] Scheff S. W., Rabchevsky A. G., Fugaccia I., Main J. A., Lumpp J. E. (2003). Experimental modeling of spinal cord injury: characterization of a force-defined injury device. *Journal of Neurotrauma*.

[33] Knerlich-Lukoschus F., Juraschek M., Blömer U., Lucius R., Mehdorn H. M., Held-Feindt J. (2008). Force-dependent development of neuropathic central pain and time-related CCL2/CCR2 expression after graded spinal cord contusion injuries of the rat. *Journal of Neurotrauma*.

[34] Knerlich-Lukoschus F., Noack M., von der Ropp-Brenner B., Lucius R., Mehdorn H. M., Held-Feindt J. (2011). Spinal cord injuries induce changes in CB1 cannabinoid receptor and C-C chemokine expression in brain areas underlying circuitry of chronic pain conditions. *Journal of Neurotrauma*.

[35] Knerlich-Lukoschus F., von der Ropp-Brenner B., Lucius R., Mehdorn H. M., Held-Feindt J. (2011). Spatiotemporal CCR1, CCL3(MIP-1alpha), CXCR4, CXCL12(SDF-1alpha) expression patterns in a rat spinal cord injury model of posttraumatic neuropathic pain. Laboratory investigation. *Journal of Neurosurgery: Spine*.

[36] Bartholdi D., Rubin B. P., Schwab M. E. (1997). VEGF mRNA induction correlates with changes in the vascular architecture upon spinal cord damage in the rat. *European Journal of Neuroscience*.

[37] Lee Y. L., Shih K., Bao P., Ghirnikar R. S., Eng L. F. (2000). Cytokine chemokine expression in contused rat spinal cord. *Neurochemistry International*.

[38] McTigue D. M., Tani M., Krivacic K. (1998). Selective chemokine mRNA accumulation in the rat spinal cord after contusion injury. *Journal of Neuroscience Research*.

[39] Myer D. J., Gurkoff G. G., Lee S. M., Hovda D. A., Sofroniew M. V. (2006). Essential protective roles of reactive astrocytes in traumatic brain injury. *Brain*.

[40] Pineau I., Sun L., Bastien D., Lacroix S. (2010). Astrocytes initiate inflammation in the injured mouse spinal cord by promoting the entry of neutrophils and inflammatory monocytes in an IL-1 receptor/MyD88-dependent fashion. *Brain, Behavior, and Immunity*.

[41] Fitch M. T., Silver J. (2008). CNS injury, glial scars, and inflammation: inhibitory extracellular matrices and regeneration failure. *Experimental Neurology*.

[42] Popovich P. G., Longbrake E. E. (2008). Can the immune system be harnessed to repair the CNS?. *Nature Reviews Neuroscience*.

[43] Knerlich-Lukoschus F., Von Der Ropp-Brenner B., Lucius R., Mehdorn H. M., Held-Feindt J. (2010). Chemokine expression in the white matter spinal cord precursor niche after force-defined spinal cord contusion injuries in adult rats. *Glia*.

[44] Horner P. J., Power A. E., Kempermann G. (2000). Proliferation and differentiation of progenitor cells throughout the intact adult rat spinal cord. *The Journal of Neuroscience*.

[45] Weiss S., Dunne C., Hewson J. (1996). Multipotent CNS stem cells are present in the adult mammalian spinal cord and ventricular neuroaxis. *The Journal of Neuroscience*.

[46] Yamamoto S.-I., Yamamoto N., Kitamura T., Nakamura K., Nakafuku M. (2001). Proliferation of parenchymal neural progenitors in response to injury in the adult rat spinal cord. *Experimental Neurology*.

[47] Knerlich-Lukoschus F., Krossa S., Krause J., Mehdorn H. M., Scheidig A., Held-Feindt J. (2015). Impact of chemokines on the properties of spinal cord-derived neural progenitor cells in a rat spinal cord lesion model. *Journal of Neuroscience Research*.

[48] Luttrell L. M. (2006). Transmembrane signaling by G protein-coupled receptors. *Methods in Molecular Biology*.

[49] Lawrence D. M. P., Seth P., Durham L. (2006). Astrocyte differentiation selectively upregulates CCL2/monocyte chemoattractant protein-1 in cultured human brain-derived progenitor cells. *Glia*.

[50] Kriegstein A. R., Götz M. (2003). Radial glia diversity: a matter of cell fate. *Glia*.

[51] McDermott K. W., Barry D. S., McMahon S. S. (2005). Role of radial glia in cytogenesis, patterning and boundary formation in the developing spinal cord. *Journal of Anatomy*.

[52] Pinto L., Götz M. (2007). Radial glial cell heterogeneity-The source of diverse progeny in the CNS. *Progress in Neurobiology*.

[53] Rakic P. (1972). Mode of cell migration to the superficial layers of fetal monkey neocortex. *Journal of Comparative Neurology*.

[54] Vaccarino F. M., Fagel D. M., Ganat Y. (2007). Astroglial cells in development, regeneration, and repair. *Neuroscientist*.

[55] Hasegawa K., Chang Y.-W., Li H. (2005). Embryonic radial glia bridge spinal cord lesions and promote functional recovery following spinal cord injury. *Experimental Neurology*.

[56] Shibuya S., Miyamoto O., Itano T., Mori S., Norimatsu H. (2003). Temporal progressive antigen expression in radial glia after contusive spinal cord injury in adult rats. *Glia*.

[57] Virgintino D., Errede M., Rizzi M. (2013). The CXCL12/CXCR4/CXCR7 ligand-receptor system regulates neuro-glio-vascular interactions and vessel growth during human brain development. *Journal of Inherited Metabolic Disease*.

[58] Abbadie C. (2005). Chemokines, chemokine receptors and pain. *Trends in Immunology*.

[59] Abbadie C., Bhangoo S., de Koninck Y., Malcangio M., Melik-Parsadaniantz S., White F. A. (2009). Chemokines and pain mechanisms. *Brain Research Reviews*.

[60] Boddeke E. W. G. M. (2001). Involvement of chemokines in pain. *European Journal of Pharmacology*.

[61] Clark A. K., Old E. A., Malcangio M. (2013). Neuropathic pain and cytokines: current perspectives. *Journal of Pain Research*.

[62] White F. A., Miller R. J. (2010). Insights into the regulation of chemokine receptors by molecular signaling pathways: functional roles in neuropathic pain. *Brain, Behavior, and Immunity*.

[63] White F. A., Wilson N. M. (2008). Chemokines as pain mediators and modulators. *Current Opinion in Anaesthesiology*.

[64] Verge G. M., Milligan E. D., Maier S. F., Watkins L. R., Naeve G. S., Foster A. C. (2004). Fractalkine (CX3CL1) and fractalkine receptor (CX3CR1) distribution in spinal cord and dorsal root ganglia under basal and neuropathic pain conditions. *European Journal of Neuroscience*.

[65] Clark A. K., Yip P. K., Grist J. (2007). Inhibition of spinal microglial cathepsin S for the reversal of neuropathic pain. *Proceedings of the National Academy of Sciences of the United States of America*.

[66] Clark A. K., Yip P. K., Malcangio M. (2009). The liberation of fractalkine in the dorsal horn requires microglial cathepsin S. *Journal of Neuroscience*.

[67] Austin P. J., Kim C. F., Perera C. J., Moalem-Taylor G. (2012). Regulatory T cells attenuate neuropathic pain following peripheral nerve injury and experimental autoimmune neuritis. *Pain*.

[68] Luongo L., Sajic M., Grist J., Clark A. K., Maione S., Malcangio M. (2008). Spinal changes associated with mechanical hypersensitivity in a model of Guillain-Barré syndrome. *Neuroscience Letters*.

[69] Chen G., Park C., Xie R., Berta T., Nedergaard M., Ji R. (2014). Connexin-43 induces chemokine release from spinal cord astrocytes to maintain late-phase neuropathic pain in mice. *Brain*.

[70] Gao Y.-J., Ji R.-R. (2010). Chemokines, neuronal-glial interactions, and central processing of neuropathic pain. *Pharmacology and Therapeutics*.

[71] Nakagawa T., Kaneko S. (2010). Spinal astrocytes as therapeutic targets for pathological pain. *Journal of Pharmacological Sciences*.

[72] Hubscher C. H., Johnson R. D. (2006). Chronic spinal cord injury induced changes in the responses of thalamic neurons. *Experimental Neurology*.

[73] Zhao P., Waxman S. G., Hains B. C. (2007). Modulation of thalamic nociceptive processing after spinal cord injury through remote activation of thalamic microglia by cysteine-cysteine chemokine ligand 21. *Journal of Neuroscience*.

[74] Jalalvand E., Javan M., Haeri-Rohani A., Ahmadiani A. (2008). Stress- and non-stress-mediated mechanisms are involved in pain-induced apoptosis in hippocampus and dorsal lumbar spinal cord in rats. *Neuroscience*.

[75] Corasaniti M. T., Amantea D., Russo R., Bagetta G. (2006). The crucial role of neuronal plasticity in pain and cell death. *Cell Death and Differentiation*.

[76] Benamar K., Geller E. B., Adler M. W. (2008). First in vivo evidence for a functional interaction between chemokine and cannabinoid systems in the brain. *Journal of Pharmacology and Experimental Therapeutics*.

[77] Benamar K., Geller E. B., Adler M. W. (2008). Elevated level of the proinflammatory chemokine, RANTES/CCL5, in the periaqueductal grey causes hyperalgesia in rats. *European Journal of Pharmacology*.

[78] Szabo I., Chen X. H., Xin L. (2002). Heterologous desensitization of opioid receptors by chemokines inhibits chemotaxis and enhances the perception of pain. *Proceedings of the National Academy of Sciences of the United States of America*.

[79] Chen X., Geller E. B., Rogers T. J., Adler M. W. (2007). Rapid heterologous desensitization of antinociceptive activity between mu or delta opioid receptors and chemokine receptors in rats. *Drug and Alcohol Dependence*.

[80] Zhang N., Inan S., Cowan A. (2005). A proinflammatory chemokine, CCL3, sensitizes the heat- and capsaicin-gated ion channel TRPV1. *Proceedings of the National Academy of Sciences of the United States of America*.

[81] Zhang N., Rogers T. J., Caterina M., Oppenheim J. J. (2004). Proinflammatory chemokines, such as C-C chemokine ligand 3, desensitize *μ*-opioid receptors on dorsal root ganglia neurons. *Journal of Immunology*.

[82] Anthony D. C., Couch Y. (2014). The systemic response to CNS injury. *Experimental Neurology*.

